# Antibody Dynamics of 2009 Influenza A (H1N1) Virus in Infected Patients and Vaccinated People in China

**DOI:** 10.1371/journal.pone.0016809

**Published:** 2011-02-09

**Authors:** Ming Wang, Jun Yuan, Tiegang Li, Yang Liu, Jibin Wu, Biao Di, Xi Chen, Xinhong Xu, Enjie Lu, Kuibiao Li, Yanhui Liu, Yejian Wu, Xiongfei Chen, Peng He, Yulin Wang, Jianhua Liu

**Affiliations:** Guangzhou Center for Disease Control and Prevention, Guangzhou, People's Republic of China; University of Cape Town, South Africa

## Abstract

**Background:**

To evaluate the risk of the recurrence and the efficiency of the vaccination, we followed-up antibody responses in patients with the 2009 pandemic H1N1 influenza and persons who received the pandemic H1N1 vaccine in Guangzhou China.

**Methods:**

We collected serum samples from 129 patients and 86 vaccinated persons at day 0, 15, 30, 180 after the disease onset or the vaccination, respectively. Antibody titers in these serum samples were determined by haemagglutination inhibition (HI) assay using a local isolated virus strain A/Guangdong Liwan/SWL1538/2009(H1N1).

**Results:**

HI antibody positive rate of the patients increased significantly from 0% to 60% at day 15 (χ^2^ = 78, *P*<0.001) and 100% at day 30 (χ^2^ = 23, *P*<0.001), but decreased significantly to 52% at day 180 (χ^2^ = 38, *P*<0.001), while that of vaccinated subjects increased from 0% to 78% at day 15 (χ^2^ = 110, *P*<0.001) and 81% at day 30 (χ^2^ = 0.32, *P* = 0.57), but decreased significantly to 34% at day 180 (χ^2^ = 39, *P*<0.001). Geometric mean titers (GMT) of HI antibodies in positive samples from the patients did not change significantly between day 15 and day 30 (T = 0.92, *P* = 0.36), but it decreased significantly from 80 at day 30 to 52 at day 180 (T = 4.5, *P*<0.001). GMT of vaccinated persons increased significantly from 100 at day 15 to 193 at day 30 (T = 4.5, *P*<0.001), but deceased significantly to 74 at day 180 (T = 5.1, *P*<0.001). Compared to the patients, the vaccinated subjects showed lower seroconversion rate (χ^2^ = 11, *P*<0.001; χ^2^ = 5.9, *P* = 0.015), but higher GMT (T = 6.0, *P*<0.001; T = 3.6, *P* = 0.001) at day 30 and day 180, respectively.

**Conclusion:**

Vaccination of 2009 influenza A (H1N1) was effective. However, about half or more recovered patients and vaccinated persons might have lost sufficient immunity against the recurrence of the viral infection after half a year. Vaccination or re-vaccination may be necessary for prevention of the recurrence.

## Introduction

A pandemic influenza A (H1N1) virus spread worldwide since April 2009, resulting in more than 16,000 deaths until March 2010. On 10 August 2010, WHO Director-General Dr Margaret Chan announced that the H1N1 influenza virus has moved into the post-pandemic period [1]. Although 2009 pandemic influenza A (H1N1) has been controlled, its recurrence cannot be excluded yet [2]. Guangzhou, the capital city of Guangdong province in south China, is one of the earliest attacked areas by 2009 pandemic influenza A (H1N1) virus. An inactivated vaccine against 2009 pandemic influenza A (H1N1) virus had been urgently manufactured to be used as an economical and effective weapon for the prophylaxis. To evaluate the risk of the recurrence and the efficiency of the vaccination, we conducted a follow-up study by detecting serum specimens collected from virus infected cases in an outbreak of a boarding school and vaccinated people in Guangzhou. The antibody dynamics characteristics would provide useful information for evaluating risk of the potential recurrence and efficacy of the vaccination.

## Methods

### Study Design

We investigated antibody responses in 129 patients with the pandemic influenza HIN1 and 86 persons who received the pandemic H1N1 vaccine in Guangzhou China. Patients who showed influenza symptoms, temperature ≥37.5° and viral RNA and/or antibody seroconversion for the pandemic virus were recruited in an outbreak of 2009 pandemic influenza H1N1 in a boarding school from August 21^st^ to October 15^th^, while vaccinated study subjects were recruited from healthy persons who received the vaccine provided by Ministry of Health of China on October 30^th^ 2009. These patients or vaccinated persons showed antibody negative to the pandemic virus (HI titer <1∶20) at the onset day of the disease or when they received the vaccination (day 0). Serum samples were collected from these patients and vaccinated persons at day 0, 15, 30, 180 after the onset of the disease or the vaccination, respectively. The ages of the study subjects in patient group were from 14 to 20 years, and that in vaccinated people were from 19 to 57 years. There are 86 males and 43 females in the patient group and 46 males and 40 females in vaccinated group.

The influenza A/H1N1 monovalent, split-virus, non-adjuvanted vaccines were manufactured by Tianyuan Bio-Pharmaceutical Co., Ltd. (batch number 20090902) through the nationwide vaccination program. Each dose of 0.5 ml product contained 15 µg hemagglutinin as prescribed by national guidelines. The vaccine was administered through intramuscular injection in the deltoid muscle.

### Ethics Statement

This study was approved by the ethics committee of the Guangzhou Center for Disease Control and Prevention and written informed consent was obtained from the study subjects.

### Laboratory Methods

#### Real-time RT-PCR

The existence of pandemic influenza H1N1/2009 virus was detected by real-time RT-PCR as described previously [3]. Briefly, viral RNA was extracted from 140 µL nasopharyngeal specimen using the QIAamp® Viral RNA Mini Kit (Qiagen, Cat# 52906) following the manufacturer instructions. Viral RNA copies were determined by real-time one-step RT-PCR assay using Invitrogen SuperScript™III Platinum® One-Step Quantitative Kit (Invitrogen, Cat# 11732-088) and primers/probe which sequences provided by WHO, i.e. forward primer 5′-GTG CTA TAA ACA CCA GCC TYC CA-3′, reverse primer 5′-CGG GAT ATT CCT TAA TCC TGT RGC-3′, and probe 5′-(FAM)CA GAA TAT ACA TCC RGT CAC AAT TGG ARA A (TAMRA)-3′. Reactions were first incubated at 50°C for 30 min, denatured at 95°C for 2 min, and then were thermal-cycled for 40 cycles (95°C for 15 sec, 55°C for 30 sec). Serially diluted positive viral RNA controls were used as calibrators in each run.

#### Hemagglutination Inhibition (HI) Assay

Antibody titers in these serum samples were determined by haemagglutination inhibition (HI) assay as described previously [3], [4]. The serum samples were treated with receptor destroying enzyme (RDE) from Vibrio cholerae (Denka Seiken, Cat#370013) for 18 h at 37°C and then were heat-inactivated at 56°C for 30 min according to WHO's standard procedure. Serum samples were diluted in serial two-fold dilutions from 1∶20 to 1∶640 and then mixed with 1% suspension of chicken red blood cells and 4 hemagglutinating units of a local isolated virus strain A/Guangdong Liwan/SWL1538/2009(H1N1). Specific positive antiserum and negative serum controls were included in the assay.

All the experiments were manipulated in the same laboratory of Guangzhou center for disease control and prevention.

### Statistical Analysis

Data were analyzed using EpiInfo version 3.3.2 (CDC, USA) and SPSS version 13.0 (SPSS Inc., USA). The statistically significant criterion was P-value <0.05. We compared the seroconversion rate (HI ≥40) by χ^2^-test between day 15 and day 30 or day 30 and day 180 or between natural infection and vaccinated group. We compared geometric mean titers (GMT) of those with positive results by Independent-Samples T test between day 15 and day 30 or day 30 and day 180 or between natural infection and vaccinated group. For GMT calculations, antibody levels below the detection limit (<1∶20) were assigned the value of 1∶10.

## Results

### Antibody seroconversion rate

**Figure 1:**
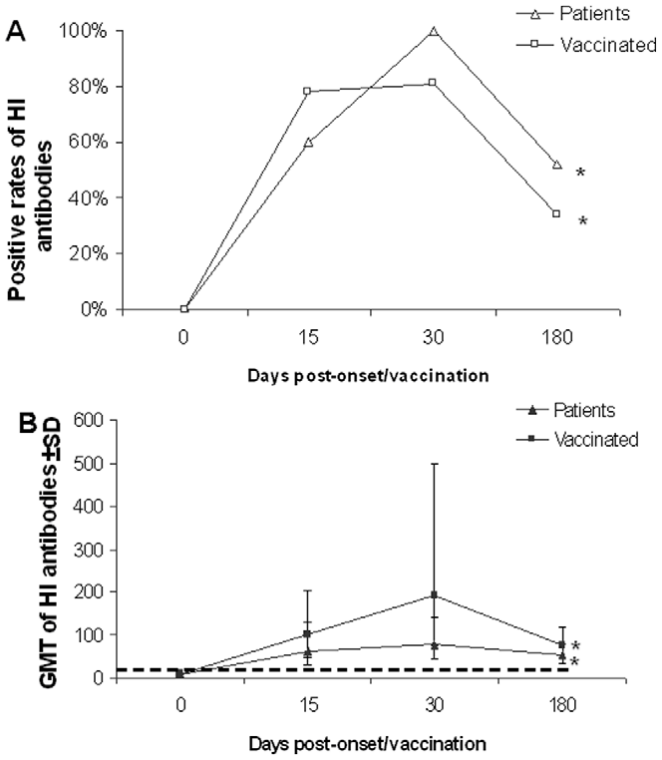
HI antibody dynamics in infected patients and vaccinated people. Note: Detection limitation (HI titer <20) is indicated by the dotted line. Error bar indicates ± standard deviation (SD) from different individual study subjects. * indicates significant differences (*P*<0.01) between results of day 30 and day 180.

HI antibody positive rate of the patients increased significantly from 0% to 60% (95%CI: 26–88%) at day 15 (χ^2^ = 78, *P*<0.001) and 100% (95%CI: 93–100%) at day 30 (χ^2^ = 23, *P*<0.001), but decreased significantly to 52% (95%CI: 42–62%) at day 180 (χ^2^ = 38, *P*<0.001), while that of vaccinated subjects increased from 0% to 78% (95%CI: 68–86%) at day 15 (χ^2^ = 110, *P*<0.001) and 81% (95%CI: 72–89%) at day 30 (χ^2^ = 0.32, *P* = 0.57), but decreased significantly to 34% (95%CI: 24–45%) at day 180 (χ^2^ = 39, *P*<0.001) (Table 1). Compared to the patients, the vaccinated subjects showed lower seroconversion rate (χ^2^ = 11, *P*<0.001; χ^2^ = 5.9, *P* = 0.015) at day 30 and day 180, respectively (Fig. 1a). In the vaccinated group, we did not find a significant difference in antibody seroconversion rate in different age groups (Table 2).

**Table 1:** Positive rate and GMT of 2009 H1N1 virus antibody in a follow-up study of natural infection patients and vaccinated people, Guangzhou, 2009.

Group	total	positive rate					GMT				
		day15	day30	day180	χ^2^		day15	day30	day180	T value	
					day15 vs day30	day30 vs day180				day15 vs day30	day30 vs day180
natural infection	129	60	100	52	23**	38**	63	80	52	0.92	4.5**
vaccination	86	78	81	34	0.32	39**	100	193	74	4.5**	5.1**

*p<0.05,

**p<0.01.

**Table 2:** Positive antibody and GMT in different age group of vaccinated people.

Age group	T15						T30						T180					
	total	Positive result			GMT		total	Positive result			GMT		total	Positive result			GMT	
		No.Positive	Positive Rate%	95%CI	GMT	95%CI		No.Positive	Positive Rate%	95%CI	GMT	95%CI		No.Positive	Positive Rate%	95%CI	GMT	95%CI
19-	30	26	87	69–96	131	81–159	30	25	83	65–94	184	121–280	28	12	43	25–63	76	56–101
30-	22	17	77	55–92	98	69–139	22	19	86	65–97	207	118–361	21	3	14	3–36	127	47–343
40-	24	17	71	49–87	94	68–130	24	20	83	63–95	204	146–286	23	9	39	20–62	69	48–98
50–60	10	7	70	34–93	80	47–135	10	6	60	26–88	160	72–355	10	4	40	12–74	57	30–107

### Geometric mean titers (GMT)

No significant difference was found in GMT of HI antibodies in positive samples collected from the patients between 63 (95%CI: 30–135) at day 15 and 80 (95%CI: 68–93) at day 30 (T = 0.92, *P* = 0.36), but it decreased significantly to 52 (95%CI: 47–58) at day 180 (T = 4.5, *P*<0.001). GMT of vaccinated persons increased significantly from 100 (95%CI: 84–120) at day 15 to 193 (95%CI: 154–242) at day 30 (T = 4.5, *P*<0.001), but deceased significantly to 74 (95%CI: 63–89) at day 180 (T = 5.1, *P*<0.001) (Table 1). Compared to the patients, the vaccinated subjects showed higher GMT at day 30 (T = 6.0, *P*<0.001) and day 180 (T = 3.6, *P* = 0.001), respectively (Fig. 1b). In the vaccinated group, we did not find a significant difference in GMT between different age groups (Table 2).

## Discussion

The 2009 pandemic influenza A (H1N1) virus is a completely new infectious agent to human being. To investigate antibody dynamics which induced by natural infection or vaccination of the virus, we conducted this prospective study by following-up the infected patients and vaccinated people in the same city for six months.

Consistent with previous reports [5]–[7], our results demonstrated that the vaccination was effective. The antibody seroconversion rate in vaccinated people achieved to a stable level more rapidly than naturally infected patients (15 vs 30 days), which might be due to the virus needs time to replicate after the infection. However, only about 80% in vaccinated people acquired protective antibody, whereas all subjects (100%) in the group of infected patients yield protective antibody at 30 days after the natural infection. It has been reported that seroconversion rates of vaccinations with live virus vaccines reached 86–97% but that of vaccination with inactivated vaccines achieved only 50–80% [8]–[10]. Our results supported that live virus vaccines may be more effective than inactivated vaccines. Furthermore, we found that higher GMT of antibody was achieved in the vaccinated subjects than in the infected patients. Previous studies also reported that GMT in recipients of live attenuated influenza vaccines was lower than that in recipients of inactivated vaccines [11], [12]. In this regard, a boost dose may be help to improve protective antibody response for live virus vaccination.

In contrast with previous studies which reported that the positive rate and GMT in children or elder persons were usually lower than the other age groups [13], we did not find a significant difference in antibody seroconversion rate and GMT levels in different age groups of vaccinated people. This might be due to that the age of study subjects recruited in this study were ranged from 19 to 60 years, so that children and elder persons did not include.

In this study, we first reported that both positive rate and GMT of antibodies to 2009 pandemic influenza A (H1N1) virus were decreased quickly in the patients and vaccinated persons. Antibody positive rates had been dropped down from 100% and 81% at day 30 to 52% and 34% at day 180, while GMTs were decreased from 80 and 193 at day 30 to 52 and 74 at day 180 in patients and vaccinated people, respective. The observation of low level antibody responses to the pandemic influenza viral infection and quick decrease of protective antibody levels may be able to explain why some pandemic influenza patients acquired a re-infection shortly as reported by Perez et al [14]. Considering the antibody levels tend to further decrease subsequently, these recovered patients and vaccinated persons may probably have no sufficient immunity against the recurrence of the viral infection. Thus, vaccination or re-vaccination may be necessary for prevention of the recurrence.

## References

[1] WHO (2010). In focus: H1N1 now in the post-pandemic period.. http://www.who.int/csr/disease/swineflu/en/.

[2] WHO (2010). http://www.who.int/csr/disease/swineflu/7th_meeting_ihr/en/.

[3] Wang M, Di B, Zhou DH, Zheng BJ, Jing H (2006). Food markets with live birds as source of avian influenza.. Emerg Infect Dis.

[4] Wang M, Fu CX, Zheng BJ (2009). Antibodies against H5 and H9 avian influenza among poultry workers in China.. N Engl J Med.

[5] Girard MP, Tam JS, Assossou OM, Kieny MP (2010). The 2009 A (H1N1) influenza virus pandemic: A review.. Vaccine.

[6] Plennevaux E, Sheldon E, Blatter M, Reeves-Hoché MK, Denis M (2009). Immune response after a single vaccination against 2009 influenza A H1N1 in USA: a preliminary report of two randomised controlled phase 2 trials.. Lancet.

[7] Greenberg ME, Lai MH, Hartel GF, Wichems CH, Gittleson C (2009). Response to a monovalent 2009 influenza A (H1N1) vaccine.. N Engl J Med.

[8] Bernstein DI, Zahradnik JM, DeAngelis CJ, Cherry JD (1982). Clinical reactions and serologic responses after vaccination with whole-virus or split-virus influenza vaccines in children aged 6 to 36 months.. Pediatrics.

[9] Belshe RB, Gruber WC, Mendelman PM, Cho I, Reisinger K (2000). Efficacy of vaccination with live attenuated, cold-adapted, trivalent, intranasal influenza virus vaccine against a variant (A/Sydney) not contained in the vaccine.. J Pediatr.

[10] Kilbourne, D E (1976). Comparative efficacy of neuraminidase-specific and conventional influenza virus vaccines in induction of antibody to neuraminidase in humans.. J Infect Dis.

[11] Sasaki S, Jaimes MC, Holmes TH, Dekker CL, Mahmood K (2007). Comparison of the influenza virus-specific effector and memory B-cell responses to immunization of children and adults with live attenuated or inactivated influenza virus vaccines.. J Virol.

[12] Clements ML, Murphy BR (1986). Development and persistence of local and systemic antibody responses in adults given live attenuated or inactivated influenza A virus vaccine.. J Clin Microbiol.

[13] Zhu FC, Wang H, Fang HH, Yang JG, Lin XJ (2009). A novel influenza A (H1N1) vaccine in various age groups.. N Engl J Med.

[14] Perez CM, Ferres M, Labarca JA (2010). Pandemic (H1N1) 2009 reinfection, Chile.. Emerg Infect Dis.

